# Essential Public Health Services’ Accessibility and its Determinants among Adults with Chronic Diseases in China

**DOI:** 10.1371/journal.pone.0125262

**Published:** 2015-04-23

**Authors:** Miaomiao Tian, Heng Wang, Xuetao Tong, Kun Zhu, Xiaojuan Zhang, Xi Chen

**Affiliations:** 1 Institute of Medical Information, Center for Health Policy and Management, Chinese Academy of Medical Sciences, Beijing, 100020, China; 2 The First Affiliated Hospital of Anhui Medical University, Hefei, 230022, Anhui, China; 3 Guiyang Medical University, Guiyang, 550004, Guizhou, China; 4 School of Management, Jiangsu University, Zhanjiang, 212013, Jiangsu, China; School of Public Health, Zhejiang University, CHINA

## Abstract

**Background:**

Along with three years implementation of health reform in China, this study aimed at providing the up-to-date evidence about the accessibility of essential public health services (EPHS) among adults with chronic diseases (CDs) in both urban and rural areas, as well as determinants in access to EPHS.

**Methods:**

The data were collected from a cross-sectional survey conducted in 2013, which used a multistage stratified random sampling method to select 54 urban communities and 54 rural villages. Hypertension patients and diabetes patients were the target population who are the main beneficiaries of EPHS. Single factor analysis of influencing factors on difference access to EPHS was performed by Chi-Square analysis. Logistic regression analysis was used to determine the predictors of effective management and effective control.

**Results:**

Patients with hypertension or diabetes were predominantly middle-aged or older persons and had a mean age of 65.26 year. People with CDs in China have a higher basic accessibility rate in EPHS with more than 90% of them having experience in receiving EPHS. And those who are willing to receive services from doctors have the most positive influence on effective management and control in blood pressure or blood glucose. But unsatisfied quality and equity of EPHS still exist in primary health system. 90% of participants could receive EPHS, but just 44% of them could control their diseases effectively. And participants from cities had the higher rates in effective management (urban: rural = 57%: 50.6%) and effective control (urban: rural = 39.5%: 27.8%).

**Conclusion:**

People with CDs have a high level in geography and economic accessibility to EPHS, but the effectiveness of health management also needs to be improved, especially for those living in rural areas. Our study highlights the continuing need for improving ability to provide EPHS and the equality among regions. Meanwhile, strengthen health education and promotion for patients with CDs to improve their willingness to receive EPHS is also highlighted.

## Background

Public health is collective actions for sustained population-wide health improvement that stresses sustainability of practices (i.e., the need to embed policies within supportive systems) and reduction in health inequalities. [1] In April 2009, China implemented her ambitious healthcare reform plan and invested RMB 850 billion (US$130.77 billion, RMB 6.5 per 1 US$) in the ensuing 3 years. [2] The goal of the reform is to achieve primary health for all by 2020. One of the main reform measures is to improve the utilization and availability of essential public health services (EPHS). [3] In 2009, nine EPHS including 1) provide personal health records; 2) health education and promotion; 3) health management for children aging from 0 to 36 months; 4) health management for maternal; 5) health management for the elder; 6) health management for people with hypertension or diabetes; 7) vaccination services; 8) reporting of communicable diseases; 9) health management for people with psychosis were implemented and provided for all residents in China, and the other two services were added in EPHS in 2011, which were public health emergency reporting and health inspection. [4,5] Meanwhile, the funding standard for essential public health services was increased from RMB 15 per capita (the average per capita public health funding) in 2009 to RMB 30 per capita in 2013. The government beefed up its efforts to improve residents’ health and public health services’ accessibility.

Chronic diseases (CDs) are diseases of long duration and generally slow progression that are largely preventable through the reduction of their main lifestyle risk factors, such as unhealthy diet, physical inactivity, tobacco use, and harmful use of alcohol. [6]Research shows that increasing the health awareness of chronically ill and high-risk populations by health management is the most effective way to prevent and delay the onset of CDs, which not only helps people know more about diseases and increases their ability for self-care, but also helps them to quit unhealthy behaviors and lead healthy lifestyles. [7,8] In China’ health reform, people with hypertension or diabetes are the main focus group of EPHS [4]. The prevalence rates of two diseases were 54.9‰ and 10.7‰ respectively according to the Fourth National Health Services Survey in China. [9] Meanwhile, providing health education and promotion, regular health checkups as well as regular follow-ups for them are the main tasks of EPHS. [10]

Primary health care institutions have the responsibilities in EPHS providing for residents and implement health management strategy for them. In China, primary health care institutions consist two parts: on the one hand are institutions in urban areas, including primary health care centers, centers for disease control and prevention. On the other hand are those in rural areas, including township hospitals, village clinics. They are the principal providers of EPHS to local residents to maintain their health. It was reported that 60% of local residents preferred to obtain health services from primary health care institutions. [11] However, the ability of urban health system and rural health system did not improve at the same pace, with urban areas on balance prospering far more than rural ones. [12,13] In 2012, the infant mortality rate in urban areas was 5.2 (per 1,000 live births), whereas it was up to 12.4 in rural areas. Moreover, there were 8.54 health professionals per 1,000 population in urban areas, but the rate in rural areas was only 3.41 in 2012 [14]. Inequalities in the accessibility of EPHS and health outcomes between rural and urban areas are still the key problems that the China Government has to face. [15] Research shows that the capabilities of health institutions determines the health level of local residents; which is influenced by disease treatment, public health services, and health promotion and management, with the latter having an enormous influence on services utilization and accessibility. [16,17]

Although researches had consider the developing gap between urban and rural areas, few investigations of the accessibility and factors contributing to EPHS among chronically ill adults mainly focused on both urban area and rural area. Along with three years implementation of health reform and the policy actions implemented in primary health care system, this study aimed at providing the up-to-date evidence about accessibility of EPHS among adults with hypertension or diabetes in both urban and rural areas, as well as the current determinants in access to EPHS.

## Method

### Ethics Statement

All of the research methods and investigational tools in this study were approved by the Ethics Committee of Chinese Academy of Medical Sciences. All of the respondents in this manuscript gave a written informed consent to participate in the study, provided consent before filling out the questionnaire, and consented to the publication of the data.

### Study population

The data were collected from a cross-sectional survey conducted from April to June 2013. A multistage stratified random sampling method was used in this study. Administration division of China consists of five practical levels of local government: the province, prefecture (city), county (district), township (subdistrict), and village (community).[18] The provincial administrative regions of China have been divided into three groups based on administrative regionalism and economic development, which are Eastern China, Central China and Western China.[19] One province was randomly selected from each region as samples, with the results of Jiangsu (Eastern region), Hubei (Central region), and Chongqing (Western region).

In each province, 3 cities were selected according to the development level. Following the sequence of district-block-residential area, two districts in urban area and two counties in rural area were selected from each city, then three communitiesapp:ds:community were selected from each district area and three counties were selected from each rural area. And one community health service station from each community and one village clinics from each county were selected. Totally, 3 provinces, 9 cities, 18 districts, 18 counties, as well as 54 communities and 54 villages were selected as the study areas.

According to the focus groups of EPHS in China, we chose 2 categories population with CDs as the research objects: hypertension patients and diabetes patients. These people are the main beneficiaries of EPHS and their utilization can reflect the implementation achievement of EPHS for those with CDs. According to the prevalence rate of hypertension (21% in 2013) and diabetes (11.6% in 2013) of China [11], 14 hypertension patients, 8 diabetes patients were randomly selected from the eligible candidates listed in health records in each community and village. Totally, 1367 hypertension patients, 806 diabetes patients responded to the study with response rates equal to 97.36%, 93.29% respectively.

### Questionnaire

The questionnaires were designed based on National Essential Public Health Services Specifications (2011)[20] and consisted of four parts: socio-demographic information, utilization of corresponding EPHS, health outcome and willingness of residents. The factors are selected according to geographical, economical and psychological accessibility of EPHS for patients. The frequencies and content of follow-ups were inquired. Participants were also asked questions about the distance to the nearest healthcare institution, willing to receive public health services. Socio-demographic information collected included gender, age, educational level, marital status, and income. The standards of Per capita net income are divided into two classes, one is the Per capita net income of urban China in 2012 (RMB 24565), and the other is Per capita net income of rural China in 2012 (RMB 7917). Health outcome mainly focus on effective control of the blood pressure of hypertension patients or blood glucose of diabetes patients.

Hypertension (or high blood pressure) is a chronic medical condition in which the blood pressure in the arteries is elevated. [21] High blood pressure is said to be present if it is often at or above 140/90 mmHg. Normal blood pressure at rest is within the range of 100–140 mmHg systolic (top reading) and 60–90 mmHg diastolic (bottom reading). [22] Diabetes is a group of metabolic diseases in which there are high blood sugar levels over a prolonged period. [23] Diabetes is characterized by recurrent or persistent hyperglycemia, and is diagnosed by demonstrating as fasting plasma glucose level ≥ 7.0 mmol/l (126 mg/dl) or plasma glucose ≥ 11.1 mmol/l (200 mg/dl) two hours after a 75g oral glucose load as in a glucose tolerance test. Normal fasting plasma glucose is within the range of 3.9–6.1 mmol/l and normal plasma glucose <7.8 mmol/l. [24] Defined in National Essential Public Health Services Specifications (2011), effective control of hypertension or diabetes means that blood pressure at rest or blood glucose level is kept in normal range for at least 3 months. [20]

### Statistical methods

Duplicate data entry method was adopted for entering all the data into the EpiData Info version 3.1 database and statistics program (Atlanta, Georgia, USA). Data entry screens were used to revise incorrect entries (e.g. logical errors, input errors). Statistical analysis was performed in SPSS statistics 12.0 (SPSS, Chicago, IL, USA).

Single factor analysis of influencing factors on difference access to EPHS was performed by Chi-Square analysis. Binary logistic regression analysis was used to conduct the multivariate analysis. Independent variables are socio-demographic characteristics (area, gender, age, marital status, education level income), distance to the nearest healthcare institution and willing to receive health services. Dependent variables are effective health management (regular follow-ups) which reflect the utilization of corresponding EPHS; and effective blood pressure/glucose control of people with hypertension or diabetes which reflect the health outcome. Effective health management is defined as patients received four or more times follow-ups from doctors in a year in National Essential Public Health Services Specifications (2011) [20]. The level of significance was set at *P* ≤0.05.

## Results

### Socio-demographic characteristics of the participants

Participants were predominantly middle-aged or older persons and had a mean age of 65.26 years (SD = 10.71 years, range = 25–95 years) with the majority being female (54.4%). 62.9% of participants were hypertension patients and 806 participants have diabetes. In all 2173 participants, 65.9% of them were classified as low income, whose personal yearly income was lower than corresponding per capita net income (rural or urban). And nearly 48% of participants were below or at elementary educational level; just 24.6% of them had an educational period in high school and above. In the aspect of geographical accessibility, 84.4% of them could reach the nearest health care institution in 1000 meters. More socio-demographic information can be found in Table 1.

**Table 1:** Socio-demographic characteristics of participants in China.

Characteristics		Participants	Percent
		(N = 2173)	(%)
Area	Urban	1150	52.9
	Rural	1023	47.1
Gender	Male	991	45.6
	Female	1182	54.4
Disease	Hypertension	1367	62.9
	Diabetes	806	37.1
Age-group	<45	109	5
	45–54	211	9.7
	55–64	569	26.2
	65–74	851	39.2
	≥75	433	19.9
Marital status	Unmarried	8	0.4
	Married	1796	82.7
	Divorced	22	1
	Windowed	347	16
Highest educational level attained	No formal education	459	21.1
	Elementary	583	26.8
	Middle school	597	27.5
	High school and above	534	24.6
Personal yearly income ^*a*^	>Per capita net income	741	34.1
	<Per capita net income	1432	65.9
Distance to the nearest health care institution	≤500m	1710	78.7
	501–1000	124	5.7
	1001–2000	267	12.3
	>2000m	72	3.3

Note: a. For rural China, the standard of Per capita net income was RMB 7917. For urban China, the standard of Per capita net income was RMB 24565.

### Accessibility in receiving essential public health services

The main duties of EPHS for residents with CDs are to implement health management for them in case of disease progression, which is an effective way to reduce the threat of diseases to people's health [25]. Among all participants, 90.29% of them had received EPHS by follow-ups or check-ups from medical technician, but near 58% of those patients had received regular follow-ups from medical technician. It showed that 53.34% of all participants were accessibility to the effective management of CDs from primary health care providers.

Table2 presents the effective management rate of patients with hypertension or diabetes under essential public health services in different characteristics. Results of univariate analyses indicated that socio-demographic characteristics including living area, education level, distance to the nearest health care institution and willing to receive services from doctors had significant relationships with effective management (p-values <0.05). It showed that more residents with hypertension or diabetes in urban areas (57%) could receive effective management from medical technician than those in rural areas (50.6%). Of the 55.7% of participant aged 75 year who got effective management was higher than that of younger people. Participants who had lower yearly income had a higher rate in receiving follow-ups regularly (55.1%). And those who live near the primary health institutions were likely to reach higher availability on getting effective management (<1000m: >2000m = 57.1%: 37.5%). What’s more, more residents who were willing to participate in health management could receive regular follow-ups (58.5%) than those who did not want to be managed (17.8%) (Table 2).

**Table 2:** Effective management rate of patients under essential public health services.

Characteristics		Ineffective group		Effective group		x^2^	*P*
		N	%	N	%		
Area	Urban	478	43	632	57	5.056	**0.025**
	Rural	505	49.4	518	50.6		
Gender	Male	440	44.4	551	55.6	1.817	0.178
	Female	559	47.3	623	52.7		
Age-group	<45	64	58.7	45	41.3	7.894	0.1
	45–54	98	46.4	113	53.6		
	55–64	258	45.3	311	54.7		
	65–74	387	45.5	464	54.5		
	≥75	192	44.3	241	55.7		
Marital status	Unmarried	4	50	4	50	0.474	0.924
	Married	820	45.7	976	54.3		
	Divorced	10	45.5	12	54.5		
	Windowed	165	47.6	182	52.4		
Highest educational level attained	No formal education	219	47.7	240	52.3	12.228	**0.023**
	Elementary	285	48.9	298	51.1		
	Middle school	271	45.4	326	54.6		
	High school and above	224	42	310	58.1		
Personal yearly income	>Per capita net income	356	48	385	52	1.94	0.164
	<Per capita net income	643	44.9	789	55.1		
Distance to the nearest health care institution	≤500m	737	43.1	973	56.9	17.552	**0.001**
	501–1000	53	42.7	71	57.3		
	1001–2000	164	61.4	103	38.6		
	>2000m	45	62.5	27	37.5		
Willing to receive services from doctors	Yes	806	41.5	1135	58.5	19.624	**0.001**
	No	193	83.2	39	17.8		

The aim of health management is to improve health. For hypertension patients and diabetes patients, effective blood pressure or blood glucose control is the main purpose. In all participants, 33.65% of them had controlled their blood pressure or glucose in the normal range for least three months. Table 3 presents the effective control rate of participants under essential public health services in different characteristics. Results of univariate analyses indicated that living area, educational level and willing to receive services from doctors had significant relationships with effective control (p-values <0.05). More residents with hypertension or diabetes living in urban areas (39.5%) could control blood pressure or glucose in normal range than those in rural areas (27.8%). And those with high educational level had a higher effective control rate (42.7%) than those who had elementary or below educational experience (31.2%). Most of participants who were not willing to receive services from doctors (92.7%) could not achieve effective control. Participants living different far from primary health care institutions had no significant difference (P>0.05). Although participants with higher income had a little lower effective control rate (34.4%) than those with lower income (36.2%), the difference between these two groups did not statistical significance (Table 3).

**Table 3:** Effective control rate of participants under essential public health services.

Characteristics		Ineffective group		Effective group		x^2^	*P*
		N	%	N	%		
Area	Urban	656	60.5	454	39.5	28.88	**0**
	Rural	739	72.2	284	27.8		
Gender	Male	627	63.3	364	36.7	0.982	0.322
	Female	772	65.3	410	34.7		
Age-group	<45	69	63.3	40	36.7	3.388	0.495
	45–54	140	66.4	71	33.6		
	55–64	377	66.3	192	33.7		
	65–74	529	62.2	322	37.8		
	≥75	284	65.6	149	34.4		
Marital status	Unmarried	6	75	2	25	2.389	0.496
	Married	1158	64.5	638	35.5		
	Divorced	11	50	11	50		
	Windowed	224	64.6	123	35.4		
Highest educational level attained	No formal education	307	66.9	152	33.1	13.119	**0.004**
	Elementary	412	70.7	171	29.3		
	Middle school	372	62.3	225	37.7		
	High school and above	306	57.3	228	42.7		
Personal yearly income	>Per capita net income	486	65.6	255	34.4	0.731	0.398
	<Per capita net income	913	63.8	519	36.2		
Distance to the nearest health care institution	≤500m	1089	63.7	621	36.3	3.359	0.339
	501–1000	82	66.1	42	33.9		
	1001–2000	175	65.5	92	34.5		
	>2000m	49	68.1	23	31.9		
Willing to receive services from doctors	Yes	1184	61	757	39	90.655	**0**
	No	215	92.7	17	7.3		

### Factors affecting EPHS’ accessibility among people with hypertension or diabetes

Variables which were statistical significance (*P* ≤0.05) in univariate analyses were tested for association with management status and control status using multivariate logistic regression respectively. In the aspect of effective management, interestingly, results indicated that perspectives of patients’ internal factors including educational expectance, age were not significantly associated with effective health management. However, living area, distance to health care institutions and willing to receive health services these external factors were the potentially modifiable factors associated with effective management. Participants living in rural China had 1.23 times the odd of receiving regular health management than those living in urban (OR = 1.232, 95% CI = 1.028–1.475, *P* = 0.024). Those living nearer to the health care institution were more likely to have effective management (≤500m OR = 2.361, 95% CI = 1.797–2.323, *P* = 0.009). And participants who were willing to receive health care from doctors had 1.27 times the odd of having effective management than those not willing to receive services(OR = 1.265, 95% CI = 0.808–1.627, *P* = 0.020) (Table 4).

**Table 4:** Multivariable analyses examining factors associated with effective management in participants.

Predictor	Reference category	B	*P*	OR	95% CI
**Area**	Urban				
Rural		0.208	**0.024**	1.232	1.028,1.475
**Education level**	No formal education				
Elementary		-0.119	0.560	0.888	0.596,1.324
Middle school		-0.196	0.306	0.822	0.564,1.197
High school and above		-0.008	0.965	0.992	0.688,1.430
**Age-group**	<45				
45–54		-0.577	0.012	0.562	0.358,0.880
55–64		-0.114	0.525	0.892	0.627,1.269
65–74		-0.004	0.976	0.996	0.766,1.295
≥75		-0.052	0.972	0.95	0.748,1.205
**Distance to the nearest health care institution**	>2000m				
≤500m		0.908	**0.009**	2.361	1.797,2.323
501–1000		0.75	**0.012**	2.117	1.166,3.728
1001–2000		0.493	0.077	1.637	0.964,2.872
**Willing to receive health care from doctors**	No				
Yes		0.213	**0.020**	1.265	0.808,1.627

In the aspect of effective control, willing to receive health care from doctors was positively associated with controlling blood pressure or blood glucose effectively, which was 9.25 times the odds of effective control than those do not want to receive health care regularly (OR = 9.252, 95% CI = 5.578–15.346, *P*<0.001). What’s more, areal variation also existed with rural residents 1.81 times the odds of effective control comparing with those living in urban. Also, participants who experienced higher education were more likely to achieve effective control than those with no formal education (Table 5).

**Table 5:** Multivariable analyses examining factors associated with effective control in participants.

Predictor	Reference category	B	*P*	OR	95% CI
**Area**	Urban				
Rural		0.593	**0.000**	1.809	1.463,2.235
**Education level**	No formal education				
Elementary		0.400	0.054	0.67	0.446,1.007
Middle school		0.608	**0.002**	1.545	0.670,1.802
High school and above		0.367	**0.039**	1.693	0.474,2.014
**Willing to receive health care from doctors**	No				
Yes		2.225	**0.000**	9.252	5.578,15.346

## Discussion

According to the results of this study, adults with hypertension or diabetes in sample regions have a higher basic accessibility rate in EPHS with more than 90% of them having experience in receiving EPHS. Accessibility can be analyzed in three aspects: geographical accessibility, economical accessibility and psychological accessibility. [26] In the aspect of geographical accessibility, just 3.3% of participants could not reach EPHS in 2000 meters, and 84.4% of them could reach the nearest health care institution in 1000 meters (around 10 minutes in walk). This should attribute to the infrastructure construction since health reform of China. Project of standardized construction of primary health institutions was one of the main duties in health reform, which had a purpose that one administrative village or community should built one village clinic or community health station. By the end of 2013, 93% of administrative villages or communities have at least one primary health institution. [27] Therefore, most of Chinese people now could reach primary health services in their living block. In the aspect of economical accessibility, personal yearly income is not a determine factor of receiving EPHS and effective control in this study (P>0.05). That’s mainly impacted by Central Government’s funding for EPHS. In 2013, the funding standard for EPHS was RMB 30 per capita and all EPHS provided for local residents are free. [28] People with hypertension or diabetes do not need pay for regular follow-ups and check-ups from primary health providers. In the aspect of psychological accessibility, participants who were willing to receive EPHS were more likely to comply with health management from primary health providers and implement self-management in control blood pressure or blood glucose. Researches show that patients had higher therapy compliance when they were willing to see physicians and obtain health services and self-care instructions from them. [29,30]

Although the creditable increase occurred in the accessibility, we found that unsatisfied effect and equity of EPHS affected the performance of primary health system. For example, among all participants, 53.8% of them could receive effective management from healthcare services’ providers, but more than 66% of all participants could not control their blood pressure or blood glucose in the normal range. Moreover, the performance of urban health system and rural health system did not improve at the same pace, with urban areas on balance prospering far more than rural ones. In this study, participants from cities had the higher rates in CDs’ effective management (urban: rural = 57%: 50.6%) and effective control (urban: rural = 39.5%: 27.8%). There were 8.54 health professionals per 1,000 population in urban areas, but the rate in rural areas was only less than 4 (3.41 in 2012). [31] Theoretically, EPHS should be provided proactively by health providers in primary health institutions. However, due to the shortage of human resources, the services of follow-up were normally provided though residents visiting the primary health institutions instead of home visit by health providers. Researches showed that the long distance and high travel cost could be the obstacles for local residents getting EPHS, especially those in rural areas [32]. Although the government has identified training new physicians as a top priority and plans to train 300,000 new physicians over the next ten years, expanding the team of public healthcare providers in primary health system has been difficult. [33] Moreover, it is reported that nearly 80% of village physicians are below vocational educational level, and 5% of them practice without a degree. [29] Many of them practice in a low level of medical technology. Insufficient and low-competence human resource caused the problem of inequality and inadequate of public health services. [34] The human resource related inequality of public health services is another problem that the health reform has to face.

According to our findings, those who are willing to receive services from doctors have the most positive influence on effective management and control in blood pressure or blood glucose. Patients who do not want to receive services regularly mainly due to two reasons. First is that CDs have not affected their normal life yet and they do not pay much attention on CDs. Meanwhile, residents' health consciousness is still weak. Especially those living in rural areas are lack of CDs’ knowledge and self-management instructions. [29] Low-literacy situation of local residents is one of the obstacles of effective implementation of health education and promotion. [35–36] The second reason is that many of them do not believe in the capability of diagnosis and treatment of EPHS’s providers, cause of the low-competence human resource. [34] Researches showed that people with CDs who needed therapy and treatment are more likely to find therapist in tertiary hospitals rather than doctors in primary health institutions in China. [37]

## Limitation

The sample in this study consists of adults with hypertension or diabetes, which may or may not represent the population with CDs. Our data of distance to the nearest health care institution and willing to receive services from doctors were based on self-report, which may be subject to respondent bias. Another potential limitation of our research is the accessibility analysis (geographical, economical and psychological accessibility) just related to two results indexes of whether followed up for 4 times per year and whether hypertension or diabetes were well controlled according to the EPHS items for those with hypertension or diabetes, which may or may not narrowed the universality of results.

## Conclusion

Adults with CDs have a higher level in geography and economic accessibility to EPHS. Although all residents with hypertension or diabetes could receive EPHS from primary health care institution, the accessibility of effective health management were also needed to be improved, especially those living in rural areas. Our study highlights the continuing need for human resource construction in primary health care institutions, which have the major responsibility to provide effective and equal EPHS for residents. China’s public health system should be intensively improved in the aspects of ability to provide EPHS and the equality among regions. Meanwhile, Patients’ willingness level to receive EPHS determines their acceptance initiative of services. Strengthen health education and promotion for patients with CDs to improve their willingness to receive EHPS is also highlighted.
